# Determination of Thermophysical Parameters Involved in The Numerical Model to Predict the Temperature Field of Cast-In-Place Concrete Bridge Deck

**DOI:** 10.3390/ma12193089

**Published:** 2019-09-22

**Authors:** Aleksandra Kuryłowicz-Cudowska

**Affiliations:** Department of Mechanics of Materials and Structures, Faculty of Civil and Environmental Engineering, Gdansk University of Technology, Narutowicza 11/12, 80-233 Gdansk, Poland; aleksandra.kurylowicz-cudowska@pg.edu.pl; Tel.: +48-58-348-6149

**Keywords:** cast-in-place concrete, temperature, boundary conditions, on-line monitoring, numerical simulations, strength, extradosed bridge, SHM system

## Abstract

The paper deals with a concept of a practical computation method to simulate the temperature distribution in an extradosed bridge deck. The main goal of the study is to develop a feasible model of hardening of concrete consistent with in-situ measurement capabilities. The presented investigations include laboratory tests of high performance concrete, measurements of temperature evolution in the bridge deck and above all, numerical simulations of temperature field in a concrete box bridge girder. A thermal conductivity equation in the author’s program, using finite difference method has been solved. New approach for identification of the model parameters and boundary conditions (heat transfer coefficients) has been proposed. The numerical results are verified by means of a wide set of experimental tests carried out on three stages of the extradosed bridge studies. A high agreement between the concrete temperature distribution in the time and space domain was obtained. The temperature history of concrete hardening, supplemented with maturity method equations, made it possible to estimate an early-age compressive strength of the cast-in-place concrete. The proposed solution could be applied in a Structural Health Monitoring system for concrete objects.

## 1. Introduction

Attention to the quality, durability and rapid construction process is one of the major challenges for the concrete industry. Prediction of the temperature field in concrete objects allows us to use the proper care of young concrete as well as to estimate development of compressive strength. In the case of massive structures, it is particularly important to not exceed the temperature difference between the interior and the concrete surface. Too high temperature gradient generates an increase in thermal-shrinkage stresses, what could be the reason of exceeding the tensile strength and as a consequence cracking occurrence [1]. Knowledge about the temperature distribution is important not only for the type of construction discussed above, but also for medium-weight and thin-walled concrete elements, for which time is a major factor to achieve the required strength to start prestressing or formwork removal. The temperature field in concrete combined with maturity method equations gives the possibility to estimate concrete strength changes over time and space [2,3].

In recent years, there has been growing interest in rationalization of the building schedule. Supporting the process of designing, curing and monitoring concrete features with the use of computer technologies, provide a lot of benefits, especially for contractors [3,4]. ElSafty et al. [5] developed the complex tool (The Deck Cracking Spreadsheet) to predict the early-age cracking of concrete bridge decks. The thermal aspect of concrete hardening is described there using time-dependent parameters. The concrete heat of hydration is calculated based on the concrete mixture proportions and the constituent material properties. The detailed input data in this tool can improve temperature forecast. However, the developed spreadsheet is limited to the decks with thickness about 20 cm and there are no analysis comparing the measured and predicted temperature evolution of concrete bridge deck [5].

The key aspect in the prediction of temperature distribution of cast-in-place (CIP) concrete is the choice of the numerical model, which should be consistent with the measurement capabilities. The multi-field mathematical models of young concrete [6,7] are well documented in a literature, but very often an application of the theoretical formulations are adopted only at the laboratory level. The major problem is that there are many constants that cannot be determined under field conditions. Therefore, the main purpose of this work is the determination of thermophysical model parameters of CIP concrete for numerical modeling of concrete temperature distribution in the bridge deck, as a part of the service offered by the Structural Health Monitoring (SHM) system.

The well-defined model parameters and initial-boundary conditions corresponding to the real environmental conditions at the construction site are the basis of reliable predictions of the in-place concrete temperature distribution. In this paper, two thermo-chemical models of hardening of concrete [8,9] were used. The heat flow equations were solved with the finite difference method in Matlab environment. The preliminary calculations of temperature evolution in bridge slab were published in the works [10,11], wherein the selection of model parameters based on the literature. A novel element of the paper is a complex proposition to identify model parameters and boundary conditions i.e., the heat transfer coefficients of free and protected concrete surfaces. Numerical simulations were confirmed by concrete temperature data measured on the construction site of extradosed bridge located in Poland. An own monitoring system was applied to register the advancement of hydration reaction of high performance concrete, C 60/75 class. Based on the temperature field and augmented maturity method, the dates of prestressing of individual sections of bridge deck, was also determined. 

## 2. Multifield Modeling of Early-Age Concrete 

The plurality and unrecognizability of phenomena occurring during concrete curing, leads to extensive experimental research and the development of a model, in which concrete is defined as a material with non-stationary properties. Mathematical models, available in the literature, takes into account four aspects of concrete hardening. It can be said that in the early age concrete there is a strong coupling of thermal, chemical, moisture and mechanical phenomena. In this study complex models presented i.e., in Azenha’s [6,12] and Di Luzio-Cusatis’s [7] papers were considered. However, having in mind the application of model to on-line monitoring of the structure under the construction process and limited number of measurement parameters, the thermo-chemical models described below, in Section 2.1 and Section 2.2, have been chosen.

### 2.1. Cervera’s et al. Model

The process of cement hydration is related to the change of the phase composition of the medium. Phase transitions are accompanied by thermal phenomena associated with the heat release, as a result of hydration reactions of cement components. As a reason of heat conduction, the temperature field appears in every area of the body. In the macroscopic approach, concrete can be treated as an isotropic and homogeneous material with constant conductivity (λ=const.)

**Figure 1:**
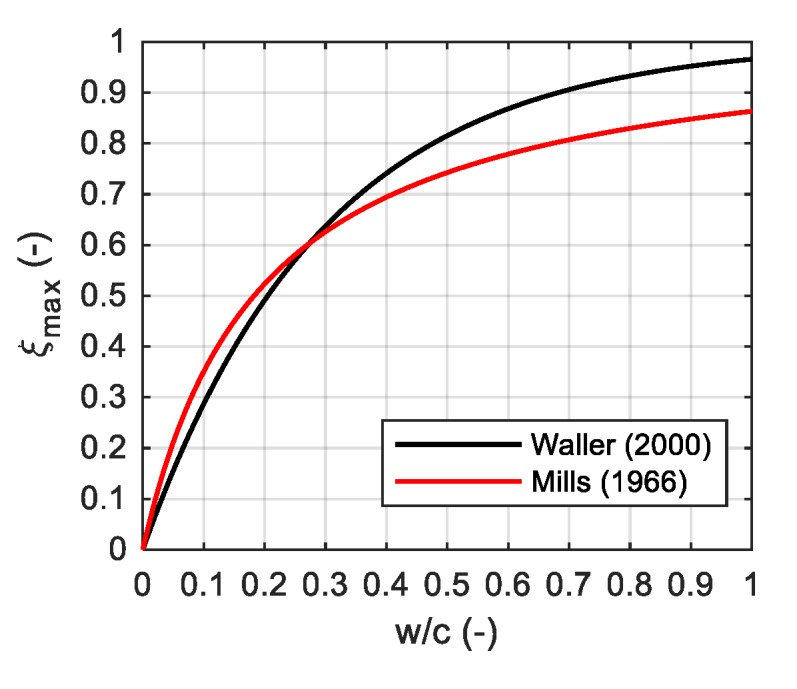
Final hydration degree $\xi_{\max}$ versus w/c ratio.

The model proposed by Cervera et al. [8] consists of two coupled equations: Thermal (1) and chemical kinetic equation, expressed as a function of evolution of hydration degree (2):

(1) $$\nabla \cdot \lambda \nabla T+Q_{\xi }\dot{\xi }=\rho c\dot{T},$$

(2) $$\dot{\xi }=\tilde{A}(\xi )\exp\left(-\frac{E_{a}}{R\cdot T}\right),$$

where the following parameters are responsible for heat transfer: Concrete density ρ, specific heat of concrete c, thermal conductivity λ, material constant $Q_{\xi }$ (heat of cement hydration in concrete), activation energy $E_{a}$ and gas constant R. In this model, a normalized internal variable, i.e., a degree of hydration ξ, which evolution allows us to predict the advancement of the hardening process has been introduced. Due to the thermally activated nature of cement hydration, a strong dependence ξ on temperature T is observed, and the Arrhenius type law is responsible for the kinetics of these changes. The normalized chemical affinity $\tilde{A}(\xi )$ is expressed through the chemical affinity $\bar{A}$ and permeability η [8]. Hence, the rate of hydration $\dot{\xi }$ can be finally obtained:

(3) $$\dot{\xi }=\underset{\tilde{A}(\xi )=\tilde{A}\eta \;(normalised\;chemical\;affinity)}{\underset{︸}{\frac{\kappa }{n_{0}}\left(\frac{A_{0}}{\kappa }\frac{1}{\xi_{\max}}+\xi \right)\left(\xi_{\max}-\xi \right)\exp\left(-\bar{n}\frac{\xi }{\xi_{\max}}\right)}}\underset{\left(Arrhenius\;typ\;law\right)}{\underset{︸}{\exp\left(-\frac{E_{a}}{R\cdot T}\right)}},$$

where $\kappa /n_{0}$, $A_{0}/\kappa$ and $\bar{n}$ are material constants. The value of the final degree of hydration $\xi_{\max}$ depends on the water-cement ratio w/c and can be determined in an approximate way, using the formula suggested by Mills [13]:

(4) $$\xi_{\max}=(1.031\cdot w/c)/(0.194+w/c),$$

or Waller [14]:

(5) $$\xi_{\max}=1-1/\exp(3.38(w/c-\delta )),$$

where without fly ash and silica fume additives δ=0. A comparison of both approaches is presented in Figure 1. Two propositions give similar results for the ratio w/c<0.27. However, the above formulas do not include the type and fineness of cement.

The normalized chemical affinity is directly measurable during an adiabatic test in which heat is not exchanged with the environment, thus the Equation (1) is simplified to:

(6) $$Q_{\xi }\dot{\xi }=\rho c\dot{T},$$

At the end of an adiabatic test $T=T_{\max}$ and $\xi =\xi_{\max}$. Marking the initial temperature of concrete by $T_{0}$, it is possible to express a $Q_{\xi }$ constant as:

(7) $$Q_{\xi }=\rho c\;(T_{\max}-T_{0})/\xi_{\max},$$

By substituting the Equation (2) and (7) into (6) we get:

(8) $${\tilde{A}}_{test}=\frac{\xi_{\max}\dot{T}}{\left(T_{\max}-T_{0})\exp(-E_{a}/(R\cdot T)\right)},$$

Formula No. 8 allows us to calculate the normalized affinity by measuring the temperature rate $\dot{T}$ during the adiabatic calorimetric test. Parameters $\kappa /n_{0}$, $A_{0}/\kappa$ and $\bar{n}$ can be determined from the regression analysis by fitting the function $\tilde{A}(\xi )$ to the results of the experiment $\left({\tilde{A}}_{test}\right)$ [8].

The described model [8] is suitable for both, ordinary and high performance concrete, and it’s capabilities are presented by a wide set of experimental studies (application on the viaduct bridge, located between Denmark and Sweden).

### 2.2. Martinelli’s et al. Model

The model presented in the work of Martinelli et al. [9,15] is also a thermo-chemical model, however, it differs from the previous original proposition to simulate the temperature evolution of concrete in adiabatic and non-adiabatic conditions.

The cement hydration is the reason of concrete hardening, so the concrete maturity can be expressed by the degree of cement hydration, which in general, is defined as the ratio between the amount of hydrated cement at time t to the total amount of cement contained in the mixture. However, this is not evident due to the fact that there is no experiment that shows directly the degree of hydration. Byfors [16] presents five different definitions of the degree of hydration, adapted to specific research possibilities. According to one of them and to Martinelli’s work [9], the degree of hydration ξ(t) is expressed by the formula:

(9) $$\xi (t)=Q(t)/Q_{\max},$$

where Q(t) is the heat of hydration produced at time t, and $Q_{\max}$ means the total amount of heat. The generated heat depends on the temperature, which, in turn, is determined by the size of the sample and the boundary conditions. The analytical relationship between the degree of hydration ξ(t) and the corresponding temperature increase, in the ideal adiabatic conditions $\Delta T_{a}(t)$ is expressed by the following relationship:

(10) $$\Delta T_{a}(t)=\frac{C}{\rho c}Q_{a}(t)=\frac{C}{\rho c}\xi (t)Q_{\max},$$

where C is the cement content per 1 m^3^ volume. Based on the results of adiabatic experiments on concrete samples, the evolution of the hydration heat can be approximated using equation [9,17]:

(11) $$Q_{a}(t)=Q_{\max}\xi_{\max}e^{-{\left(\frac{a}{t}\right)}^{b}},$$

where a and b control the shape of the function.

In real structures, setting and hardening of concrete in non-adiabatic conditions take place, which generates non-stationary temperature field. In general, heat transfer through a solid can be described by the Fourier equation 1. The rate of heat source of cement hydration in concrete $\left(Q_{\xi }\dot{\xi }\right)$ expressed in equation 1 is described by the formula [9]:

(12) $$q_{c}(x,t)=C\frac{dQ_{c}(t)}{dt}.$$

The current value of the concrete temperature has a significant effect on the rate of heat source $q_{c}$, while the temperature depends on the produced heat. Therefore, the feedback effect between $q_{c}$ and T occurs. The temperature influence on the rate of chemical reactions is given by the relation:

(13) $$k(T)=A_{k}\exp\left(-\frac{E_{a}}{R\cdot T}\right).$$

**Figure 2:**
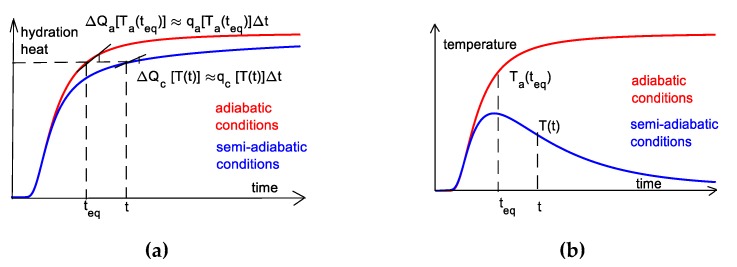
Adiabatic and semi-adiabatic conditions: (**a**) Hydration heat and (**b**) temperature development [9].

Arrhenius constant $A_{k}$ and apparent activation energy $E_{a}$ can be determined by measuring the reaction rate k(T) versus curing temperature. American standard ASTM C1074 [2] provides procedures for the calculation of mentioned values. The Arrhenius equation 13 is useful to represent the relationship between the actual heat source $q_{c}(T)$ and the corresponding one $q_{a}(T_{a})$, measured in adiabatic conditions at the same stage of reaction (Figure 2a). If concrete hardening in non-adiabatic conditions has reached the degree of hydration ξ(t) at time t, it is possible to define an equivalent time $t_{eq}$, in which the same degree of hydration will be achieved under adiabatic conditions $\left(\xi_{a}(t_{eq})=\xi (t)\right)$ [9]. Thus, it will be assumed that $Q_{a}(t_{eq})=\xi (t)\cdot Q_{\max}$. An equivalent time $t_{eq}$ may be computed from Equation (11):

(14) $$t_{eq}=a/{\left(-\ln\left(\frac{\xi (t)}{\xi_{\max}}\right)\right)}^{\frac{1}{b}}.$$

Although the heat released in non-adiabatic conditions $Q_{c}(T(t))$ at time t is equal to the heat generated in the adiabatic process $Q_{a}(T_{a}(t_{eq}))$ at time $t_{eq}$, the temperature values T(t) and $T_{a}(t_{eq})$ are not equal (Figure 2b). Considering the above dependence, the ratio of heat source of cement hydration in concrete, in both indicated conditions, can be expressed using Equation (13):

(15) $$\frac{q_{c}(T(t))}{q_{a}(T_{a}(t_{eq}))}=\frac{e^{-\frac{E_{a}}{R\cdot T(t)}}}{e^{-\frac{E_{a}}{R\cdot T_{a}(t_{eq})}}}=e^{-\frac{E_{a}}{R}\frac{T_{a}(t_{eq})-T(t)}{T_{a}(t_{eq})\cdot T(t)}}.$$

Thus, describing the heat source $q_{c}(T(t))$ using the formula 15, the partial differential equation representing the thermal energy balance in one-dimensional space is equal to:

(16) $$\lambda \cdot \frac{\partial^{2}T}{\partial x^{2}}+q_{a}(T_{a}(t_{eq}))\cdot e^{-\frac{E_{a}}{R}\frac{T_{a}(t_{eq})-T(t)}{T_{a}(t_{eq})\cdot T(t)}}=\rho c\frac{\partial T}{\partial t}.$$

Equation (16) with the defined initial-boundary conditions allows us to determine the temperature distribution of the concrete in non-adiabatic conditions.

In analyzed paper [9], the validation of the mathematical model was carried out using 150 mm cubic samples and prismatic specimens. The experimental and numerical results showed a high compatibility and the possibility to estimate the compressive strength of concrete.

## 3. Initial-Boundary Conditions

Assuming that the model describes the temperature development immediately after concrete mixing and placing, the initial condition can be written in the following form:

(17) $$T(x,y,z;t)=T_{0}(x,y,z),$$

where $T_{0}$ is the initial temperature of concrete.

**Figure 3:**
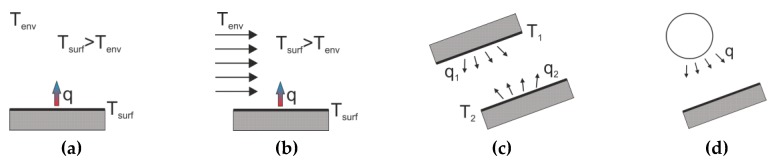
Heat transfer between a solid and the environment: (**a**) Natural convection; (**b**) forced convection; (**c**) longwave radiation and (**d**) shortwave radiation [12].

When considering boundary conditions, two mechanisms of heat transfer between a solid and the environment should be distinguished: Convection (natural and forced) and radiation (Figure 3). In natural convection, the heat flow from the concrete surface takes place as a result of the difference between the concrete and ambient temperature. In the process of forced convection, an additional factor accelerates the heat exchange, e.g., the action of wind. The second mechanism of heat transport is radiation. Longwave radiation applies to any solid substance that emits and receives radiation from the environment. Shortwave radiation is a type of energy transmission usually considered in relation to the energy emitted by the sun. It needs to be marked, that when the steel formwork is used, it can absorb large energy from the sun. As a result, the temperature of concrete can be significantly affected by the conduction heat between the steel formwork and concrete. 

The equation of the energy balance is supplemented by the Newton’s [18] or Stefan-Boltzmann’s condition. From the physical point of view, heat exchange between the surface and the environment takes place, both, through convection and radiation. Formally, the heat transfer coefficient is a convective type factor [18]. The heat flow coming to the concrete surface must be absorbed by the surrounding air. Irrespective of the driving force of the air movement, the convective heat transfer can be expressed by Newton’s law:

(18) $$q_{0}=\alpha \;(T_{surf}-T_{env}),$$

where α is the convective heat transfer coefficient and $T_{env}$ and $T_{surf}$ means the ambient and concrete surface temperature. Although, many theoretical attempts have been made to establish prediction equations for the heat transfer coefficient, accurate predictions are available only for very simple geometries and controlled environmental conditions. In irregular cases, such as variable external conditions and the complex geometry of engineering structures, the forecasts were limited to empirical correlations [12]. According to the Institute of Physics of the Cracow University of Technology [19], the heat transfer coefficient on the concrete surface for natural convection $\alpha_{nc}$ is 7 W/(m^2^·K). Klemczak gives the value of 6 W/(m^2^·K) [20] and also proposes a formula [21]:

(19) $$\alpha_{nc}=2.62\;{\left(T_{surf}-T_{env}\right)}^{0.25}.$$

**Figure 4:**
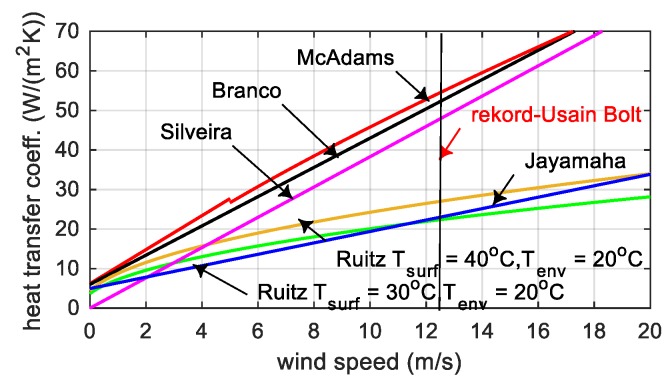
The convective heat transfer coefficient $\alpha_{fc}$ versus wind speed $v_{w}$.

For forced convection, the heat transfer coefficient on the concrete surface $\alpha_{fc}$ depends on the wind speed $v_{w}$ and can be calculated according to the McAdams [22], Jonasson [23], Branco [24], Ruitz [25], Silveira [26] and Jayamaha [27] (Figure 4). It should be emphasized that suggestions shown in Figure 4 apply to the perfect reproduction of previous tests, in other situations they can only provide approximate solutions [12].

On the construction site, an additional concrete surface insulation in the form of the styrofoam layer or foil is often used. For thermally fixed conditions, where the heat flux is perpendicular to the surface, a substitute heat transfer coefficient $\alpha_{s}$ can be determined taking into account the thickness $l_{i}$ and conduction coefficient of a particular layer $\lambda_{i}$:

(20) $$\alpha_{s}={\left(\frac{1}{\alpha }+\sum_{i=1}^{i=n}\frac{l_{i}}{\lambda_{i}}\right)}^{-1}.$$

Jonasson states that for young concrete the heat transfer coefficient is theoretically correct only for free surface, but the above formula can be applied as some approximation, when the insulation has a lower heat capacity than concrete and the volume of other materials in the formwork (plywood and styrofoam) it is small, i.e., the heat accumulated in the formwork can be omitted compared with the thermal energy in the concrete [18]. In case where there is insulation between the formwork girders, the heat flux is not perpendicular to the surface and equation 20 cannot be used [18].

## 4. Concept of a Numerical Model Consistent with the In-Situ Measurement Capabilities

The discussed models [8,9] provide very good insight into the concrete hardening process, especially for engineering applications (e.g., SHM system), where very limited data is available. The temperature of concrete and heat released during hardening are the main measurement parameters, around which hypotheses regarding the course of phenomena, mathematical theory and numerical model can be built [28,29]. Relative humidity measurements can be assessed in the surface, however, the registration of changes in concrete moisture content at any depth of the element is complicated, for example due to the construction of this type of sensor. The detection of concrete strains and microcracks could be implemented using ultrasonic methods, more and more often used successfully, but requiring extremely precise evaluation. The implementation of such an extensive system that monitors thermal, moisture and mechanical parameters at the construction site conditions, combined with numerical analysis, becomes an extremely laborious task to rival with traditional methods of thermal and mechanical properties evaluation. However, this is not an obstacle to integrate the possibility of thermal changes registration in a structure with numerical verification and prediction of temperature distribution in the time and space domain.

The thermal aspect of early age concrete should focus on the integral parameters, used by engineers, during controlling the hardening process. These parameters should have a clear technical sense, be easily measurable in the monitoring system and have their equivalents in the mathematical model. The main thermal parameters are the:

- Initial temperature of the mixture (°C),
- Ambient temperature (°C),
- Temperature at the concrete surface (°C),
- Maximum temperature of concrete and time of its occurrence (°C), (h),
- Maximum increase of temperature, as a difference between maximum temperature and initial temperature (°C),
- Maximum rate of temperature increase determined as the quotient of the temperature difference in the period of its increase up to time of this period (°C /h),
- Spatial temperature gradient defined as the quotient of the maximum temperature difference measured at the same time in two selected points,
- Released heat of cement hydration calculated based on the approximate formulas.

In addition, it is necessary to control the wind speed (m/s), the thickness of the element (m), type of formwork and insulation.

In the function of the above parameters, simulations of the temperature field in the concrete bridge deck were performed. For this purpose, two thermo-chemical models presented in Cervera’s [8] and Martinelli’s [9] papers were used. Although the mentioned models take into account only thermal and chemical aspects, its implementation for real structures is a difficult task. The discussed models simplify the problem of heat transfer omitting the heat movement by migrating moisture and assuming that the heat transport only by conduction is carried out. According to many authors, the influence of moisture diffusion on heat movement in concrete is not significant [30,31].

Prediction of temperature field of CIP concrete requires knowledge of the temperature development of concrete in adiabatic conditions. Due to the difficulties in the availability of such commercial devices, a novel method for determination of adiabatic hydration curve was proposed. To identify this curve, the concrete temperature measurements inside 150 mm cubic samples should be taken. These specimens are curing under isothermal conditions, i.e., in a water bath at constant temperature and under semi-adiabatic conditions in styrofoam containers, especially prepared for this purpose. The suggested approach and equations described in Martinelli’s paper [9] allow us to plot the adiabatic hydration curve and then to identify three material parameters: $\kappa /n_{0}$, $\bar{n}$ and $A_{0}/\kappa$, necessary in the mathematical model described by Cervera et al. [8]. Parameters obtained in laboratory conditions are only a reference point for the considered concrete, because they strongly depend on the geometry and atmospheric conditions prevailing at the construction site. Therefore, in the present work, for the analyzed concrete deck of the extradosed bridge, the own functions for $\kappa /n_{0}$ and $\bar{n}$ were defined., and 

The initial condition of heat transfer phenomenon does not cause many difficulties, because the initial temperature of the mixture delivered on the building site is usually known. Definitely, more complications generate the determination of boundary conditions, which constantly change under field tests (ambient temperature, wind speed, curing activities and insolation). Mainly two possibilities of heat exchange between the concrete surface and the environment deal with analyzed box bridge girder. The surface of the plate can be free or protected by a shuttering layer. In the second variant, the heat transfer coefficient on the concrete surface protected by the formwork layer $\left(\alpha_{s}{}^{form}\right)$ depends on the difference between the initial temperature of the mixture $\left(T_{0}\right)$ and the ambient temperature $\left(T_{env}\right)$. In turn, for the convective heat transfer coefficient for the free surface $\left(\alpha_{fc}\right)$, the own pattern as a function of wind speed was proposed. The presented concept for estimation thermal changes of the concrete elements of the extradosed bridge was applied.

## 5. The Description of the Extradosed Bridge

**Figure 5:**
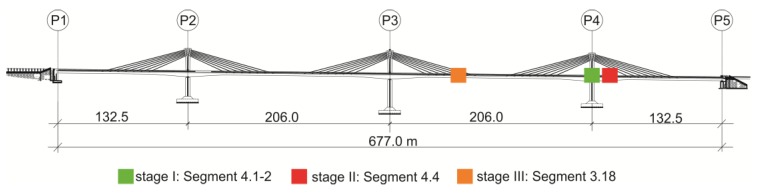
View of the object and monitored zone.

The research works on the longest span in the European extradosed bridge located in Poland were carried out (Figure 5). The aim of the studies was to determine the compressive strength of young concrete embedded in the bridge’s span in real time. Laboratory tests included compression tests of mortar and concrete samples and allowed it to develop a maturity–strength relationship for the considered concrete. Field works were focused on temperature measurements of concrete bridge deck and also on compression tests of validation specimens.

**Figure 6:**
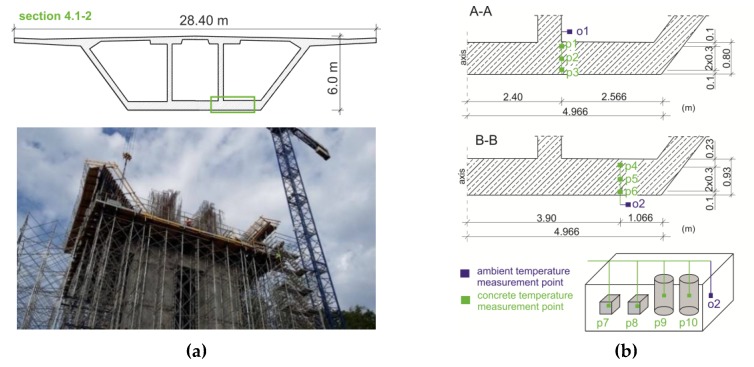
The monitored section No. 4.1–2: (**a**) View and cross section and (**b**) localization of temperature measurement points.

The bridge object (Figure 5) was monitored three times. For every considered section, the formwork of the system was made of 3-SO plywood and H20N timber formwork beams spaced at a distance of about 30 cm. The first stage involved monitoring of the bottom slab of the starting section (No. 4.1–2, Figure 6a). The temperature sensors were mounted in two cross-sections (A–A and B–B) of the bottom plate (points p1–p6). The ambient temperature was also monitored (o1, o2). In two cubes and cylinders, the temperature measurements of the concrete (p7–p10) and the measurements of the water temperature (o3) were carried out. Figure 6b illustrates the location of the measurement points.

**Figure 7:**
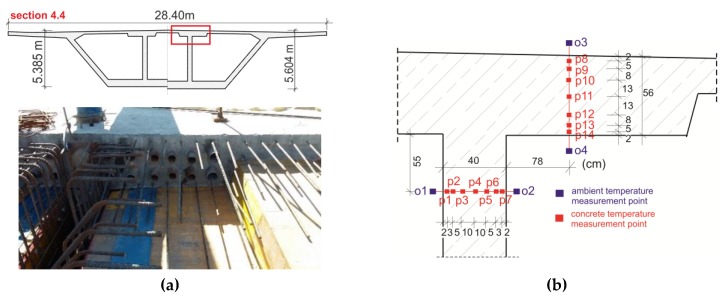
The monitored section No. 4.4: (**a**) View and cross section and (**b**) localization of temperature measurement points.

The second stage concerned the overhanging section (No. 4.4) with variable height (Figure 7a). In this case, the concrete temperature in the top slab and the web within the prestressing cables zone was monitored. This segment was concreted in two stages: The bottom plate and webs at first, and a day later, the top plate. The temperature sensors were installed in the inner web (points p1–p7) and in the top slab (p8–p14; Figure 7b). The measurements, with dedicated prefabricated modules consisting of nine sensors (seven for concrete temperature and two for air temperature) were realized. The p7 and p8 sensors were destroyed during the constructions works.

**Figure 8:**
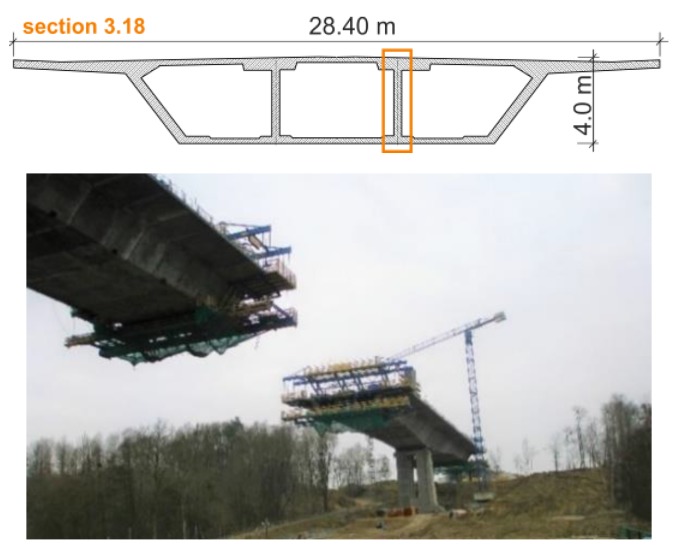
The monitored section No. 3.18.

**Figure 9:**
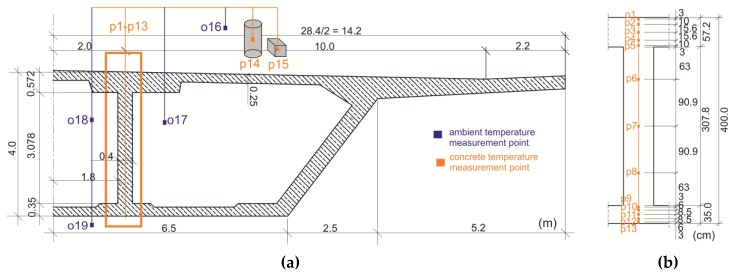
Localization of measurement points—section No. 3.18: (**a**) Global view and (**b**) detailed view.

The last, third part of the in-situ research, was dedicated to the overhanging section (No. 3.18) with a fixed height (Figure 8). The entire segment was concreted in one cycle. The temperature sensors were installed in the top plate (p1–p5), the inner web (p6–p8) and the bottom slab (p9–p13). Especially for this monitoring a prefabricated measurement module consisted of 13 sensors was designed (Figure 9b). The temperature in the cylindrical and cubic samples (p14, p15) and air temperature (o16–o19) was also registered.

Especially for field tests, the own system was designed. The recorder makes it possible to measure concrete temperature at 20 points simultaneously and works on water-resistant, digital, 1-wire sensors type ds18b20, which do not need to be calibrated. 1-wire interface requires a single digital pin, and it is possible to connect multiple ones to the same pin, each one has a unique 64-bit ID burned in at the factory to differentiate them. The ds18b20 sensor provides a temperature measurement in the range from −55 to +125 °C with an accuracy of ± 0.5 °C. The sensor is fairly precise and can give up to 12 bits of precision from the onboard digital-to-analog converter. It is powered from 3.0 V to 5.5 V. Since the sensor is digital, it does not get any signal degradation even over long distances. Measurement points can be assembled using individual sensors or by using a prefabricated measurement strip with a series of sensors. The device can be powered with a battery for about 30 days. Temperature data is transmitted via a GSM modem. 

## 6. Identification of the Model Parameters of High Performance Concrete 

### 6.1. Thermophysical Properties of C 60/75 Concrete

The tests were carried out with Portland cement CEM I 52,5N SR3/NA (Table 1), [32]. The composition of the concrete for 1 m^3^ is as follows: Cement, 440 kg; water, 143 kg; sand 0/2, 632 kg; basalt aggregate 2/8, 498 kg; basalt aggregate 8/16, 785 kg and plasticizers, 8.49 kg.

The used cement characterizes by a normal (N) initial strength gain, low content of alkali (NA: Na_2_O_eq_ < 0.6%) and very high corrosion resistance, especially sulphate (SR3: C_3_A < 3%) and chloride [33]. The considered cement is indicated for applications where a high initial and final strength is required, primarily in communication engineering and for the rapid stripping of prefabricated elements. The composition of the aggregate affects on the final strength of concrete and on the modulus of elasticity, which for concrete with basalt aggregate is about 15%–20% greater, than with granite or pebble aggregate. In the analyzed mixture, three types of admixtures: Liquefying, plasticizing and aerating were also used. The composition of the mixture was a complex process, which included selecting ingredients, performing a series of laboratory experiments and technological trials. The designed concrete had to meet the strength requirements of class C 60/75 and reach a minimum 47 MPa after 48 h. Other mix requirements were as follows: Water–cement ratio w/c<0.34, exposure class XC4 (carbonation of concrete), XD1 (chloride other than sea water) and XF2 (freeze), consistency S4 (slump range 160–210 mm) according to [34], air content 4.0%–6.0% in place, good pumpability and workability.

**Figure 10:**
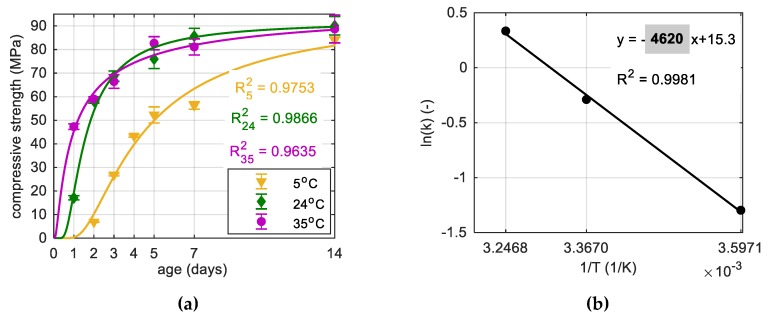
(**a**) The average compressive strength for mortar cubes and the (**b**) determination of $E_{a}/R$ [3].

In order to determine the activation energy divided by the gas constant $\left(E_{a}/R\right)$, tests on 50 mm mortar samples (acc. to ASTM C1074 [2]) were carried out. Three sets of 18 specimens were prepared and placed in a water bath at constant temperatures: $T_{w1}=5{\;}^{o}C$, $T_{w2}=24{\;}^{o}C$ and $T_{w3}=35{\;}^{o}C$. A detailed scope of testing mortar samples is described in [3]. The rate constants k for strength development and curing temperatures were determined on the basis of approximation of strength data (Figure 10a) using non-linear regression according to the equation proposed by Freiesleben Hansen et al. [35]:

(21) $$S=S_{u}\cdot \exp(-{\left(\tau /t\right)}^{\beta }),$$

where S means compressive strength at time t, $S_{u}$ is the ultimate strength, τ is the time constant and β is the shape constant. The values of τ and β were determined using approximation by the least squares method. For temperature $T_{w1}:\;\tau \;=\;3.658\;\mathrm{day},\;\beta \;=\;1.338\;(-)$, for $T_{w2}:\;\tau \;=\;1.335\;\mathrm{day},\;\beta \;=\;1.549\;(-)$ and for $T_{w3}:\;\tau \;=\;0.716\;\mathrm{day},\;\beta \;=\;0.715\;(-)$. The *k*-values for temperature 5, 24 and 35 °C equaled respectively 0.273, 0.749 and 1.397 (1/day). The results of the presented studies were $E_{a}/R=4620\;K$ (Figure 10b).

The cement content per cubic meter of concrete C, density ρ and water–cement ratio w/cbased on the composition of the mixture, was assumed. The parameters such as: $\xi_{\max}$, c and λ using literature formulas were calculated.

The final hydration degree $\xi_{\max}$ for w/c = 0.325 can be obtained from the Mills’ (4):

(22) $$\xi_{\max}=(1.031\cdot 0.325)/(0.194+0.325)=0.65,$$

and Waller’s (5) formula, where the parameter δ is equal to zero due to the absence of fly ash and silica fume:

(23) $$\xi_{\max}=1-1/\exp\;(3.38\;(0.325-0))=0.67.$$

Similar values of $\xi_{\max}$ were obtained, however, the calculations according to Mills’ proposal [13] were adopted.

**Figure 11:**
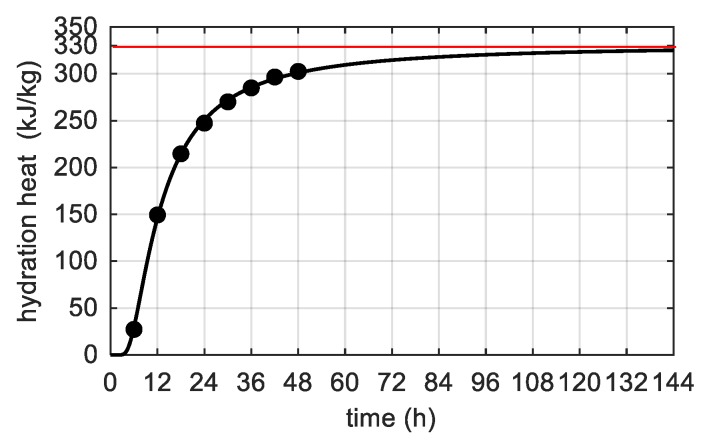
Hydration heat of CEM I 52, 5 N SR3/NA.

The hydration heat of CEM I 52,5 N SR3/NA marked in the semi-adiabatic calorimetry test given by the cement producer after 48 h was about 302 kJ/kg. In Figure 11 the extrapolated value of the total heat $Q_{\max}=330\;\mathrm{kJ}/\mathrm{kg}$ was highlighted. For one cubic meter of concrete, the $Q_{\max}$ value had to multiply by the cement content. It is also worth remarking that the considered cement was sulphate resistant (SR) and the amount of tricalcium aluminate (C_3_A) was restricted to lower than 3%, which affected the relatively low heat of cement hydration.

Prediction formulas also exist for specific heat of concrete, for instance, in the work of Lura and Breugel [36] the value of c is based on the mass component per cubic meter (W) and specific heat of each fraction of the mix (cement $c_{cem}$, basalt aggregate $c_{bas}$, quartz aggregate $c_{quar}$ and water $c_{w})$. For the degree of hydration ξ=0, the specific heat expressed in J/(kg·K) is given by relation:

(24) $$\begin{array}{l} c=\frac{W_{cem}\cdot c_{cem}+W_{bas}\cdot c_{bas}+W_{quar}\cdot c_{quar}+W_{w}\cdot c_{w}-0.2\cdot W_{cem}\cdot \xi \cdot c_{w}}{W_{cem}+W_{bas}+W_{quar}+W_{w}}=\\ \;=\frac{440\cdot 456+1283\cdot 766+632\cdot 699+143\cdot 4187}{440+1283+632+143}=890.23 \end{array},$$

and for $\xi =\xi_{\max}=0.65$ the specific heat is equal to 794.41 J/(kg·K). The high value of c reduces the extremum temperature of concrete and for a low value of specific heat, the temperature peak increases. Generally, the specific heat of concrete depends not only on the composition of the concrete mixture, but also on the temperature and humidity, which change during the hardening process. However, the models described in the literature often omit this relation and the constant specific heat estimated on the basis of the mixture composition is applied. Therefore, based on the above calculations, the model adopted the average specific heat value equals 840 J/(kg·K) = 0.84 kJ/(kg·K).

The heat conduction coefficient of concrete, expressed in W/(m·K), was estimated based on the thermal conductivities of the mix components using the following equation [36]:

(25) $$\lambda =\frac{W_{cem}\cdot \lambda_{cem}+W_{bas}\cdot \lambda_{bas}+W_{quar}\cdot \lambda_{quar}+W_{w}\cdot \lambda_{w}}{W_{cem}+W_{bas}+W_{quar}+W_{w}}=\frac{440\cdot 1.23+1283\cdot 1.91+632\cdot 3.09+143\cdot 0.6}{440+1283+632+143}=2.01$$

According to Neville [37], for concrete with basalt aggregates, this coefficient equals 2.0 W/(m·K). A similar approach has been reported by Breugel [38], which says, that the value of λ ranges between 1.9 and 2.2 W/(m·K). Thus, the computed value of the thermal conductivity is consistent with the literature proposals and for further calculations λ=2.0 W/(m·K) was assumed.

Table 2 summarizes all thermophysical parameters, fixed for the C 60/75 high-performance concrete analyzed in this paper.

### 6.2. Three Selected Parameters: $\kappa /n_{0}$, $\bar{n}$ and $A_{0}/\kappa$

**Figure 12:**
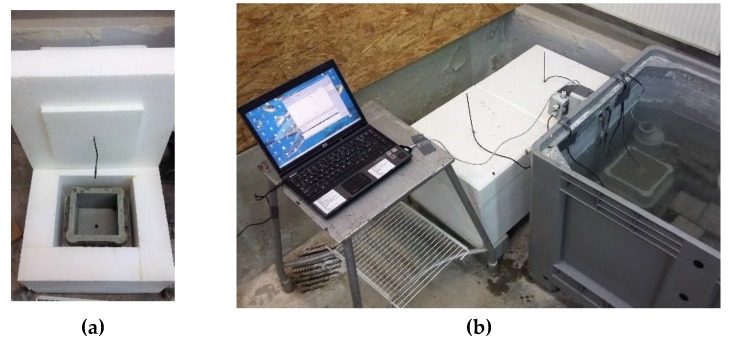
(**a**) Styrofoam container to semi-adiabatic concrete curing and (**b**) experimental set-up.

The model parameters ($\kappa /n_{0}$, $\bar{n}$ and $A_{0}/\kappa$) required to perform numerical simulations were identified experimentally. The laboratory tests involved the concrete temperature measurements in the cubic samples curing under isothermal and semi-adiabatic conditions. For this purpose, four 150 mm cubic specimens made according to the recipe of the analyzed concrete were prepared. Immediately after molding, two cubes were placed in a water bath at a constant temperature of 24 °C and the concrete temperature changes under isothermal conditions were recorded. Two more cubes were inserted in specially prepared styrofoam containers with 10 cm thick insulation layer. The insulation around the specimens slows down the rate of heat loss and limits the heat exchange with the environment, thus cubes can be cured in semi-adiabatic conditions. The measurement boxes allow free samples placement in the mold and to insert the temperature sensors (Figure 12a). The experimental set-up illustrated in Figure 12b consists of a computer, 1-wire temperature sensors, semi-adiabatic containers and a water bath.

**Figure 13:**
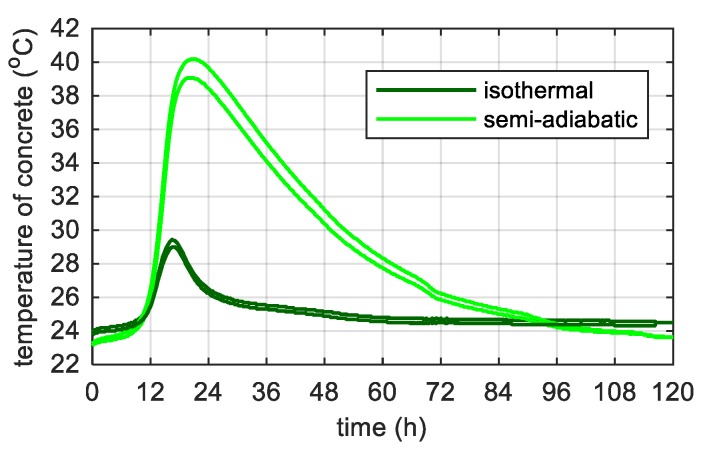
Temperature evolution in the centre of a concrete cubes cured in isothermal and semi-adiabatic conditions.

The maximum temperature in concrete cubes reached under semi-adiabatic conditions was 10.4 °C higher than in isothermal conditions and the rate of temperature increase was more than twice bigger (Figure 13). The tests have proved how large the influence of boundary conditions on the temperature is, even with such a small volume of concrete. The average characteristic values from two concrete cubes are summarized in Table 3. 

#### 6.2.1. Determination of Model Parameters Using the Martinelli’s Approach

The partial differential equation of heat flow, reported by Cervera [8] and Martinelli [9], was solved using the finite difference method in the author’s programs written in the MATLAB environment. For the models described in paragraph No. 2, the validation process was successfully performed based on literature data [8,9] and then used to analyze the own cases.

Experimental results, presented in above paragraph (Figure 13) were used to determine the adiabatic heat curve. Regression coefficients (a, b) for the function (11) and the heat transfer coefficients of the concrete hardening under isothermal ($\alpha_{nc}^{w}$) and semi-adiabatic conditions ($\alpha_{s}$) were calculated by the least squares regression. Table 4 reports the input values adopted in the model.

**Figure 14:**
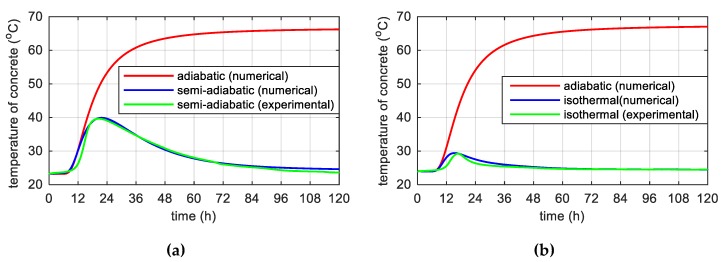
Adiabatic hydration curve for two various conditions: (**a**) Semi-adiabatic and (**b**) isothermal.

The adiabatic temperature evolution represents the response of the simulations of the semi-adiabatic and isothermal temperature development (Figure 14). In both cases, the maximum temperature of self-heating of concrete hardening in adiabatic conditions was mutually convergent and the temperature value equals:

(26) $$T_{\max}=T_{0}+\frac{C\cdot \xi_{\max}\cdot Q_{\max}}{c\cdot \rho }=23.2+\frac{440\cdot 0.65\cdot 330}{0.84\cdot 2570}=23.3+43.7=66.9\;{(}^{o}C).$$

In addition, this value was consistent with the maximum concrete temperature (67.8 °C) measured in the middle of the 93 cm thick bottom slab (point p5, Figure 6b), for which conditions close to adiabatic could be assumed. Very good agreement between real measured value of $T_{\max}$ and numerical response was confirmed.

**Figure 15:**
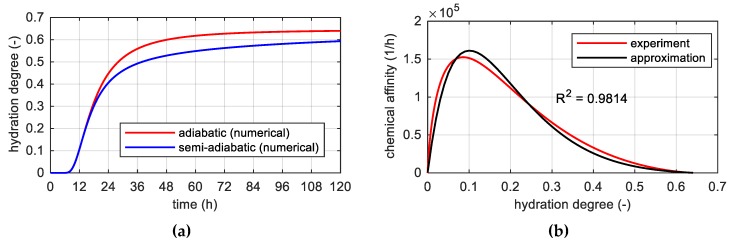
(**a**) Time evolution of the hydration degree and (**b**) chemical affinity vs. hydration degree.

The results of calculations using the finite difference method (FDM) for the considered specimens allowed us to plot the development of hydration degree ξ versus time and chemical affinity ${\tilde{A}}_{test}$ as a function of hydration degree according to equation 8, (Figure 15a). Founded on the nonlinear approximation of affinity $\tilde{A}(\xi )$ to experimental data (Figure 15b), three sought parameters of the model were obtained: $\kappa /n_{0}=6.6\cdot 10^{6}\;h^{-1}$, $\bar{n}=5.2$ and $A_{0}/\kappa =1\cdot 10^{-4}$.

#### 6.2.2. Determination of Model Parameters Using the Cervera’s Approach

**Figure 16:**
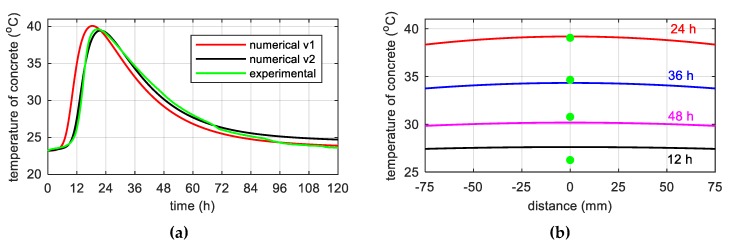
Temperature of concrete specimens cured in semi-adiabatic conditions: (**a**) In the time domain and (**b**) space variation of the temperature field in a 150-mm cube at different curing times (simulations + four measured values).

The next step included verification of the determined constants in reliance on temperature simulations of the concrete specimens according to the Cervera’s approach and also involved a possible modification of the three parameters. For this purpose two simulations were carried out: “v1” for designated constants: $\kappa /n_{0}=6.6\cdot 10^{6}\;h^{-1}$, $\bar{n}=5.2$, $A_{0}/\kappa =1\cdot 10^{-4}$ and “v2” for coefficient $A_{0}/\kappa =1\cdot 10^{-5}$. The actualization of $A_{0}/\kappa$ parameter improved the solution, which agreed with the measurement data (Figure 16).

**Figure 17:**
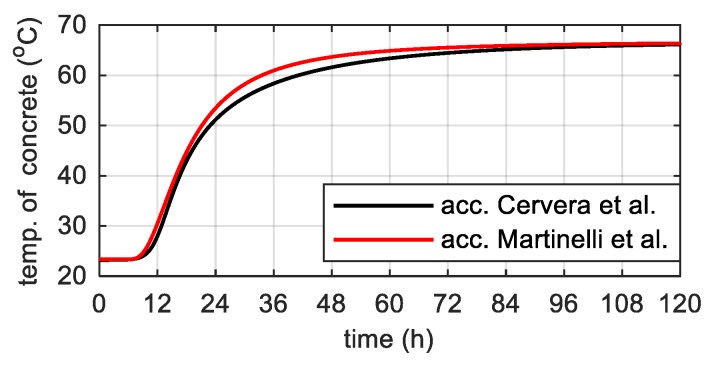
The concrete temperature in adiabatic conditions for two approaches.

The predicted adiabatic temperature evolution was consistent with Martinelli’s proposal (Figure 17). The results received in this part of the work constituted a check of the models themselves as well as determined parameters and indicated the correctness of the adopted assumptions.

Additionally, in order to recognize the influence of $\kappa /n_{0}$, $\bar{n}$ and $A_{0}/\kappa$ parameters on the temperature distribution of concrete, three cases were analyzed. In each case, one of the mentioned parameters was changed, and two subsequent remained constant:

- Case 1: Variable coefficient $\kappa /n_{0}$, fixed $\bar{n}=5.2$ and $A_{0}/\kappa =1\cdot 10^{-5}$,
- Case 2: Variable coefficient $\bar{n}$, fixed $\kappa /n_{0}=6.6\cdot 10^{6}\;h^{-1}$ and $A_{0}/\kappa =1\cdot 10^{-5}$,
- Case 3: Variable coefficient $A_{0}/\kappa$, fixed $\bar{n}=5.2$ and $\kappa /n_{0}=6.6\cdot 10^{6}\;h^{-1}$.

**Figure 18:**
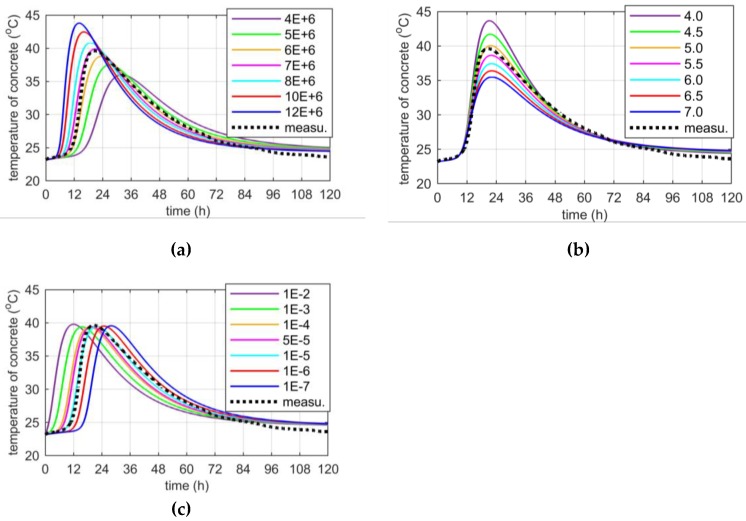
Three considered model parameters: (**a**) $\kappa /n_{0}$; (**b**) $\bar{n}$ and (**c**) $A_{0}/\kappa$.

Figure 18 shows the results of calculations for the temperature development in a concrete cubic sample. The temperature measured experimentally with a dashed, black line was marked. On the basis of Figure 18 it was found, that the coefficient $\kappa /n_{0}$ influenced the reaction rate, $\bar{n}$ was responsible for the extreme temperature value and $A_{0}/\kappa$ for the time of its occurrence. If the parameter $\kappa /n_{0}$ increased, the reaction rate also rose. The maximum peak value of concrete temperature increased every ~1 °C and time occurrence of peak was reduced twice from $\kappa /n_{0}=4\cdot 10^{6}\;h^{-1}$ to $\kappa /n_{0}=12\cdot 10^{6}\;h^{-1}$. When $\bar{n}$ rose, the extreme concrete temperature dropped gradually from 2 to 1 °C for every half from $\bar{n}=7$ to $\bar{n}=4$. If we consider coefficient $A_{0}/\kappa$ the time occurrence of peak was shorter every ~3 h form $A_{0}/\kappa =1\cdot 10^{-7}$ to $A_{0}/\kappa =1\cdot 10^{-2}$ with step equals $10^{-1}$.

## 7. Concrete Temperature Evolution of the Bridge Deck

The one-dimensional model (1D) was used to predict the temperature development of regular concrete slabs. In case of any irregularities such as the proximity of the protruding reinforcement or the influence of the temperature of earlier concreted parts of element, previously determined model parameters are no longer valid. Therefore, the calculations were performed for elements with clearly defined boundary conditions. This applies to the B-B section of the bottom plate of stage I, the web and the top plate (stage II) and the web monitored during III stage of research on the bridge.

**Figure 19:**
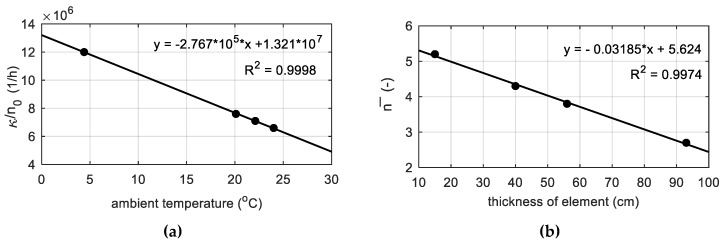
The proposition of determination model parameters: (**a**) $\kappa /n_{0}$ and (**b**) $\bar{n}$.

Numerical simulations were carried out relying on experimentally determined constants for each research stage. The selected model parameters and heat transfer coefficients were adopted from the own propositions dedicated to the analyzed high-performance concrete and element thickness up to 100 cm. In this kind of structure, semi-adiabatic conditions of concrete hardening might be assumed. The prediction of concrete temperature for compressive strength assessment is the most useful for the medium-weight structure, which constitute the building contractor’s interest. The proposed relationships are not intended for massive or thin-walled concrete elements. On the basis of laboratory and field tests, the coefficient $\kappa /n_{0}$ as a function of ambient temperature (Figure 19a), and the parameter $\bar{n}$ versus the thickness of the element (Figure 19b) was expressed. In both cases a linear function was used, and the accuracy of the approximated data (black dots) describes the determination coefficient R^2^. As shown in Figure 18c, the parameter $A_{0}/\kappa$ is mainly responsible for the time of extreme temperature occurrence, so for elements with thicknesses higher than 60 cm (80 and 93 cm) the value 1·10^−5^ was assumed, and for elements thinner than 60 cm (35, 40 and 56 cm) $A_{0}/\kappa$ equals 1·10^−4^.

**Figure 20:**
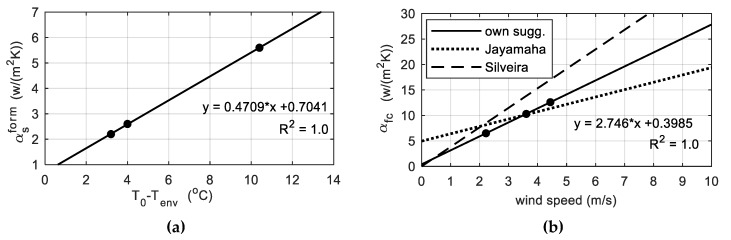
The own suggestion of connective heat transfer coefficient: (**a**) $\alpha_{s}^{form}$ and (**b**) $\alpha_{fc}^{}$.

Literature propositions for heat transfer coefficients for forced convection or for insulation layer are often the result of specific experimental investigations and are only relevant in this particular case. In this study, the convective heat transfer coefficient on the concrete surface protected by the formwork layer $\alpha_{s}^{form}$ depended on the difference between the initial temperature of the mixture $T_{0}$ and the ambient one $T_{env}$ (Figure 20a). In Figure 20 dots corresponded to the experimental data, which came from the three stages of field tests and the existing atmospheric conditions. For the heat transfer coefficient for the free surface of the slab $\alpha_{fc}^{}$, the formula as a function of wind speed was proposed. The comparison with the suggestion of other researchers is illustrated in Figure 20b.

In the future, to determine reliable nomograms (such as Figure 19 and Figure 20), a series of concrete tests should be performed for different storage conditions and different concrete volumes. This approach would be justified in the case of repetitive elements, manufactured e.g., in a prefabrication plant. However, for individual constructions, at least measurements of the temperature of concrete cubes hardening in an adiabatic calorimeter or isothermal and semi-adiabatic conditions are required. Additionally, the measurement system should be installed in one regular section on the object, e.g., the start segment.

### 7.1. Bottom Slab (Stage I)

The slab was concreted in June, when the average ambient temperature of 10 days was 22.1 °C. It was assumed that throughout the analyzed period, on the bottom surface of the slab the formwork (plywood and girders) was mounted. The top surface of the plate was exposed to the weather conditions, but between 23 and 94 h it was covered with a styrofoam layer, 5 cm thick. The average wind speed in this period was approximately 16 km/h = 4.44 m/s [39], thus according to the proposition presented in Figure 20b, coefficient $\alpha_{fc}^{}=12.6\;W/(m^{2}\cdot K)$. The initial temperature of the mixture was 26.7 °C.

**Figure 21:**
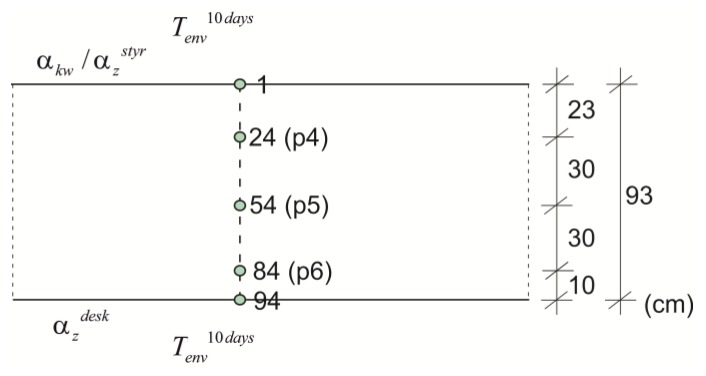
Space discretization.

**Figure 22:**
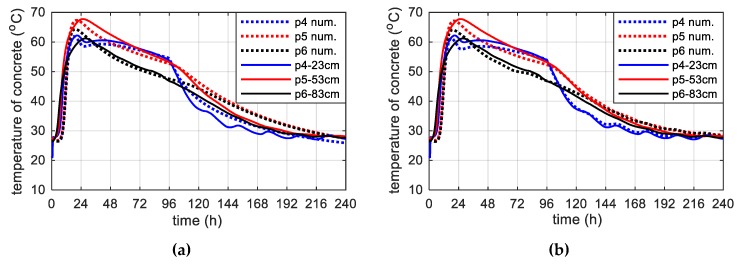
The concrete temperature of bottom slab, 93 cm thick (stage I): (**a**) Constant ambient temperature and (**b**) variable, measured ambient temperature.

**Figure 23:**
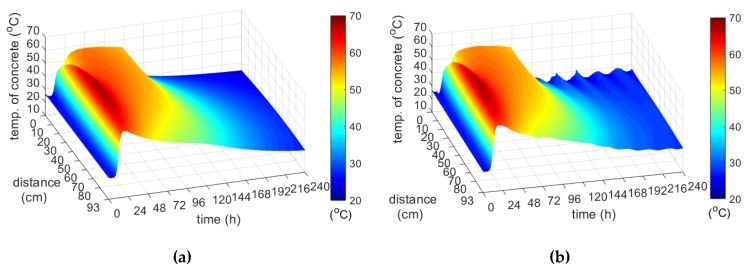
The temperature distribution of the concrete bottom slab (stage I): (**a**) Constant ambient temperature and (**b**) variable, measured ambient temperature.

Using the finite difference method, the space domain was subdivided in $m_{s}=94$ nodes whose distance Δx was 1 cm (Figure 21). Moreover, for the explicit numerical integration a time increment dt met stability criterion. The calculations were carried out for a constant (Figure 22a) and a daily time variation of ambient temperature measured over and above the concrete slab, interpolated to the adopted step dt (Figure 22b). In Figure 22, as well as in the forthcoming ones, dashed lines indicate the results from the numerical simulations, whereas solid lines correspond to the concrete temperature measured at the appropriate depths. Thermo-physical parameters were adopted according to Table 2 and Table 5. Figure 23 shows the temperature distribution, over time and plate thickness, in two variants. In Figure 23b the influence of daily temperature fluctuations can be seen, especially on the upper surface of the slab. The numerical results of temperature evolution show good agreement with the experimental data for both cases. According to Figure 22b, the relative error between measured and numerical value of maximum concrete temperature was equal to 1.6%, 0.9% and 4.5% for points p4, p5 and p6. The bigger differences concerned the time occurrence of the peak because the relative error reached 12.7%, 20.5% and 29.6%, respectively. 

The maximum concrete temperature of the bottom plate was noted after 25 h of hardening, reaching a temperature of 67.8 °C at point p5 (Figure 22). The temperature of 70 °C was not exceeded, which the standard [40] gives for the limit value. In the core of the monitored slab, the conditions close to adiabatic existed. The temperature of self-heating of concrete caused by hydration reaction was 41.1 °C. 

### 7.2. Web and Top Slab (Stage II)

**Figure 24:**
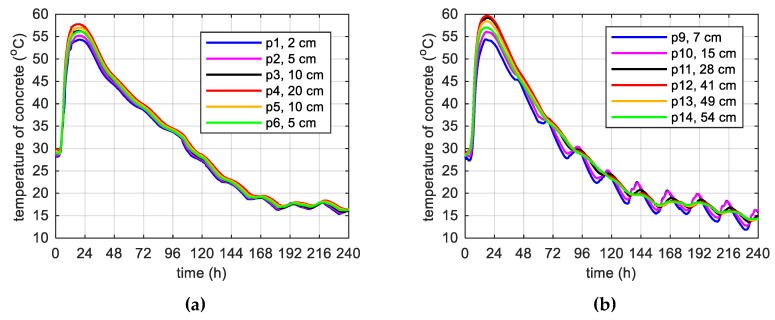
Temperature development of the concrete during 240 h: (**a**) Web and (**b**) top slab.

Another regular case is the web (40 cm) and the top slab (56 cm), i.e., medium-weight elements, monitored during the second stage of bridge investigations [11]. The recorded temperature history in the concrete web and the top plate are presented in Figure 24. The initial temperature of the mix, which was used to concrete the web was 29.1 °C, and in the case of the top plate it was 28.5 °C. The maximum concrete temperature of both the web and the top plate was recorded after 17.5 h. At point p4 and p12 its value was equal to 57.8 and 59.6 °C. Thus, the maximum temperature of self-heating caused by the cement hydration recorded in the web and the top plate was 28.7 and 31.1 °C.

In both monitored elements, boundary conditions had a strong influence on the temperature distribution. The concrete temperature of the web, 40 cm thick, surrounded on both sides with formwork, was very similar at all measurement points (Figure 24a). The top slab was on the one hand uncovered, and on the other protected with shuttering, which was the reason of temperature difference (e.g., Δp9-p12 = 5.2 °C; Figure 24b). Due to the high air temperature, these differences were not significant.

**Figure 25:**
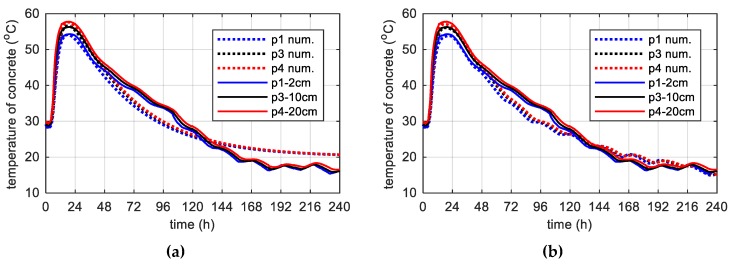
The concrete temperature of web, 40 cm thick (stage II): (**a**) Constant ambient temperature and (**b**) variable, measured ambient temperature.

**Figure 26:**
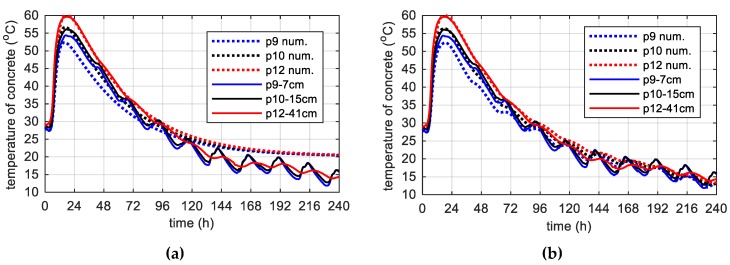
The concrete temperature of top slab, 56 cm thick (stage II): (**a**) Constant ambient temperature and (**b**) variable, measured ambient temperature.

**Figure 27:**
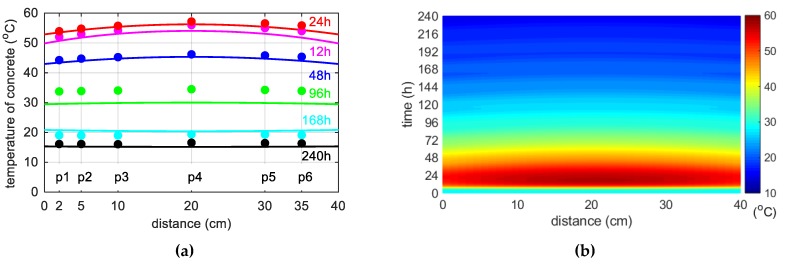
(**a**) Space variation of the web temperature field at different curing time and (**b**) concrete temperature distribution map (in both cases variable, measured ambient temperature).

**Figure 28:**
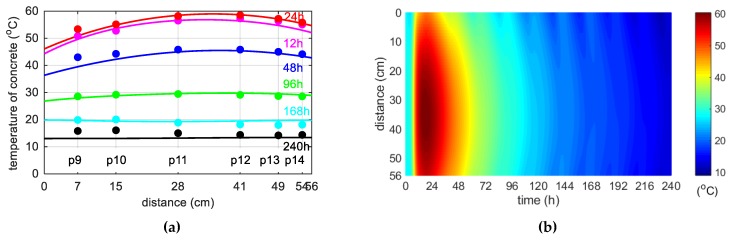
(**a**) Space variation of the top slab temperature field at different curing time and (**b**) concrete temperature distribution map (in both cases variable, measured ambient temperature).

During concreting of overhanging section of the bridge span, the average ambient temperature of 10 days was 20.1 °C. The average wind speed in this period reached 8 km/h = 2.22 m/s [39]. Calculations, as before, for constant and variable air temperature were made. Thermophysical parameters were adopted in accordance with Table 2, Table 6 and Table 7. The temperature measurements in the web and the top plate in six points were carried out, while the graph presents results from three characteristic points (due to the readability of the graph). Better convergence of numerical and experimental data was obtained in the case of using a variable ambient temperature (Figure 25, Figure 26). According to Figure 25b the relative error for points p1, p3 and p4 was equal to 0.8%, 0.3% and 1.1% (max. temperature) and 5.8%, 0.9% and 2.7% (time occurrence of max. temperature). Based on Figure 26b the relative error for points p9, p10 and p12 was equal to 3.6%, 0.5% and 0.7% (max. temperature) and 1.9%, 1.0% and 0.8% (time occurrence of max. temperature). Good results have also been achieved for the temperature distribution in the cross-section of the web and the top plate, where the measured values were marked with dots (Figure 27, Figure 28). For the web the symmetry condition of the temperature distribution was met. Conducted research confirmed correctness of model assumptions and emphasized the importance of ambient temperature.

### 7.3. Web (Stage III)

The third stage of research on the bridge object was carried out in March, when the average ambient temperature was the lowest of the considered cases and it was around 4 °C. The average wind speed was equal in this period 13 km/h = 3.61 m/s [39]. The initial temperature of the concrete mix delivered to the construction site reached the value of 14.8 °C. The maximum temperature of the concrete web was equal 34.3 °C (in point p6, p8) and it was 23.5 °C lower than the maximum temperature recorded for the same element during the second stage of testing. The lowest temperature (32.5 °C) occurred in the middle of the web height (point p7).

**Figure 29:**
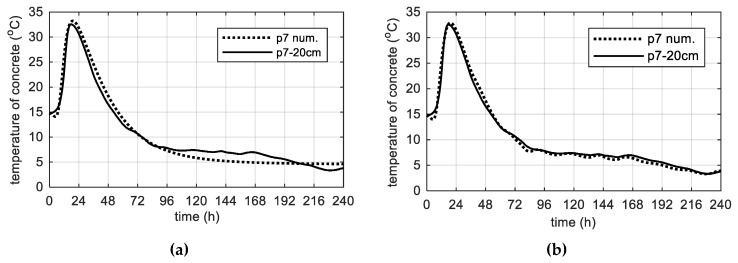
The concrete temperature of web, 40 cm thick (stage III): (**a**) Constant ambient temperature and (**b**) variable, measured ambient temperature.

As before, it was assumed, that throughout the analyzed time, the web was insulated by the formwork layer. Thermophysical parameters were adopted according to Table 2 and Table 8. The temperature measurements in the web was made along its height, therefore the least-exposed point, located in the middle of the web height (p7) was chosen. The results of numerical simulations correspond very well to the measured values (Figure 29). Based on Figure 29b, the relative error of extreme concrete temperature was equal to 1.0% and the time occurrence of peak 6.3%.

### 7.4. Limitations of One-Dimensional Approach

The proposed one-dimensional model was able to predict well both the heating and cooling phase, which is important in relation to the occurrence of undesirable tensile stresses. However, the one-dimensional model was not recommended to describe the temperature evolution of concrete plates with undefined strictly boundary conditions, as shown in the example discussed below. It concerns the bottom slab of stage I, for which the A-A measurement cross-section was located just next to the protruding web reinforcement and could not be insulated well with the styrofoam layer. The second example with irregular boundary conditions was the top and bottom plate monitored in stage No. III, where some disorder was the temperature coming from the hardening web. For these elements, the selected model parameters were modified. The obtained solutions were satisfactory, but for special cases, the calculations should be made with a two-dimensional model.

**Figure 30:**
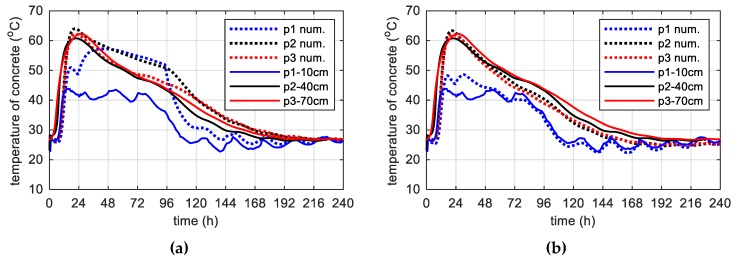
The concrete temperature of bottom slab, 80 cm thick (stage I): (**a**) Variant No. 1 and (**b**) variant No. 2.

For the bottom slab, 80 cm thick (stage I) simulations in two variants were carried out. The first one for thermophysical coefficients specified for the 93 cm slab (Table 5) with modification of $\bar{n}$parameter according to Figure 19b. The second approach with the change of heat transfer coefficients $\alpha_{s}^{styr}$ and $\alpha_{fc}$ (Table 9). The results of the calculations are shown in Figure 30. Considering the temperature distribution at the points located near the upper surface of the plate (p1, p2—Figure 30a) it could be concluded that there was no full insulation with styrofoam and the influence of protruding nearby reinforcement was substantial, hence the adopted coefficients of heat transfer $\alpha_{s}^{styr}$ and $\alpha_{fc}$ as in the case of a regular slab (93 cm, thick) was a wrong idea. Modification of these parameters improved the solution (Figure 30b).

For the A-A cross-section (80 cm thick), after 24 h, the temperature difference between points p1 and p2 was 19.3 °C (Figure 30a). Such information prompted the contractor to insulate the upper surface of the slab, which in 25–94 h was covered with a styrofoam layer, 5 cm thick. This was particularly visible in the thermal history of points located in the upper zone of the plate, i.e., p1. At this time, there was no cooling of the element, and a constant temperature was maintained.

**Figure 31:**
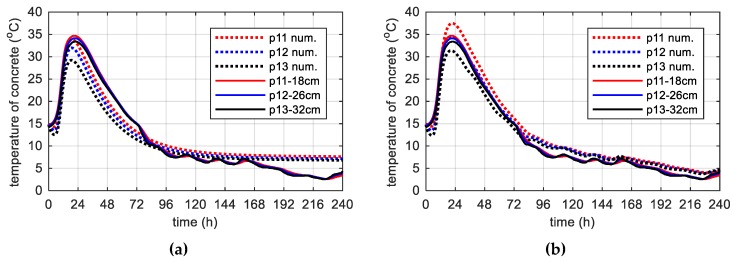
The concrete temperature of bottom slab 35 cm thick (stage III): (**a**) Constant ambient temperature and (**b**) variable, measured ambient temperature.

**Figure 32:**
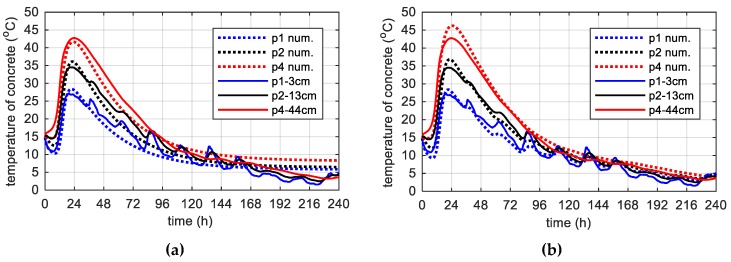
The concrete temperature of top slab 57.2 cm thick (stage III): (**a**) Constant ambient temperature and (**b**) variable, measured ambient temperature.

In stage III, the box section of the bridge was concreted in one step. Thus, for the upper surface of the bottom slab and the bottom surface of the top slab, the influence of the temperature from the embedded web was significant. Therefore, a replacement heat transfer coefficient was adopted, indicating it with a symbol $\alpha_{s}^{conc}$ (Table 10, Table 11). The others parameters, except $A_{0}/\kappa$ for the top plate and $\bar{n}$ for the bottom plate (modification $\bar{n}$ according to Figure 19b) remained unchanged relatively to the regular element for stage III, i.e., web (Table 10). For the bottom surface of the top plate the 10 days web temperature from point p6 ($T_{env\_t}^{10\;days}$) and for the upper surface of the bottom plate an average temperature from point p8 ($T_{env\_b}^{10\;days}$) was considered. The results (Figure 31 and Figure 32) were satisfying, although they required additional assumptions.

The thermal changes in the top plate differed the most (Figure 32). The max. concrete temperature occurred at point p4 was 42.8 °C. This temperature was lower by 16.8 °C than the maximum temperature recorded for the same element during the second stage of investigations. The various boundary conditions (formwork and free surface) and low ambient temperature generate difference (15.7 °C) between the center (p4) and the surface (p1) of the element. In general, guidelines state that the temperature difference must remain smaller than 15–20 °C depending on the element thickness.

The one-dimensional approach has several limitations. Early-age concrete is difficult for modeling because it is a complex material, which is additionally subjected to transformations as a result of cement hydration. The contractors use different types of formwork, insulation and the weather is changing all the time during construction process. Due to this fact, the 1–D way is not universal. Nevertheless, this fundamental method gives a possibility to control the early age behavior of concrete with quite good agreement. In the case of 1–D problem we could solve the partial differential equation using the own code of the finite difference method. There are no hardware restrictions and calculation time is short. A numerical, one-dimensional model is always a good complement to the measuring system. Many researchers [8,9,12] use this method to predict temperature evolution of concrete. 

## 8. Distribution of Concrete Compressive Strength for Selected Structural Elements of the Bridge

**Figure 33:**
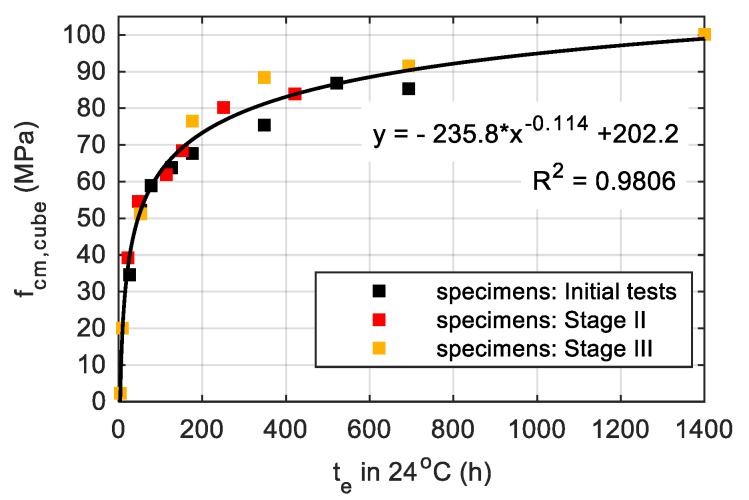
Maturity curve related to cubic strength after three stages of research.

As mentioned at the beginning of the paper, the in-place concrete temperature measurements were performed with a focus on estimation the real concrete compressive strength. The contractor planned to prestress every typical segment after reaching 60% of characteristic strength for class C 60/75, so it was exactly 45 MPa for cubic strength. Due to that fact, the maturity method was implemented [2,41]. The curve plotted after the third stage of the study is shown in Figure 33.

**Figure 34:**
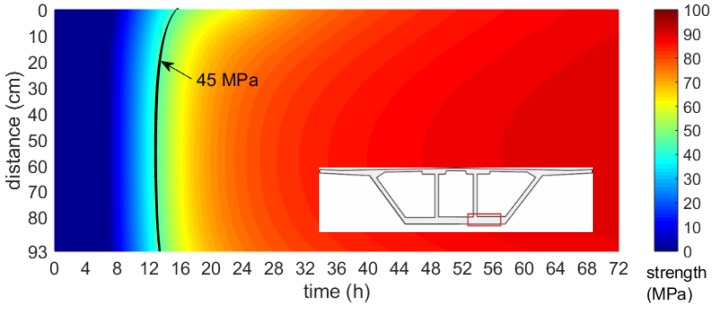
Map of the early age compressive strength distribution of the bottom slab (stage I, 93 cm thick, $T_{env}^{10\;days}=22.1{\;}^{o}C$).

**Figure 35:**
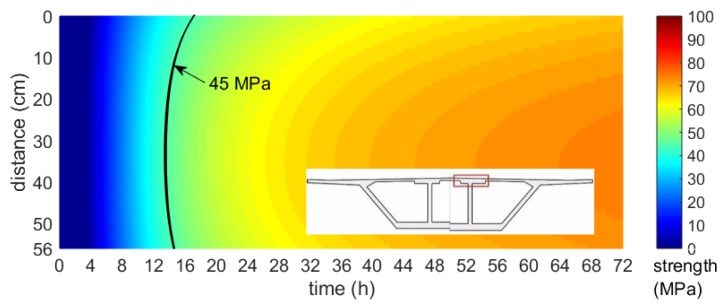
Map of the early age compressive strength distribution of the top slab (stage II, 56 cm thick, $T_{env}^{10\;days}=20.1{\;}^{o}C$).

**Figure 36:**
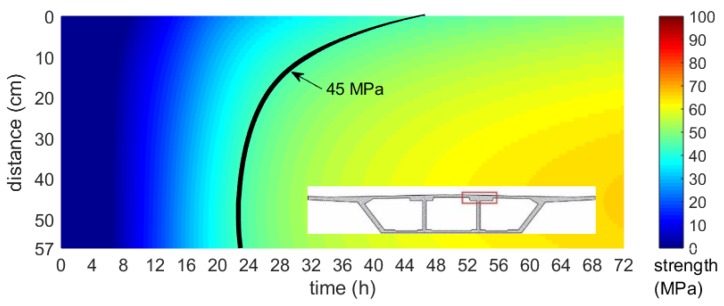
Map of the early age compressive strength distribution of the top slab (stage III, 57,2 cm thick, $T_{env}^{10\;days}=4.4{\;}^{o}C$).

Based on the numerically obtained maps of the temperature field and the maturity–strength relationship expressed in the equivalent age $t_{e}$ domain, the distribution of early concrete compressive strength was presented. In each of the considered cases, the strength was calculated according to the concrete maturity curve for cubic samples, developed after the first, second and third stage of the specimen’s validation tests performed during bridge operations. Figure 34, Figure 35 and Figure 36 illustrates the compressive strength changes of young concrete for the bottom plate (stage I) and top slabs (stage II and III) versus the first three days of hardening. It can be seen that the age needed to achieve the required strength to prestress, and differed significantly in each monitored element. For plates with the same thickness, concrete reached 45 MPa in 15–48 h (Figure 35, Figure 36). In the case of the top plate monitored during third stage of research, the desirable concrete strength in the surface zone was achieved definitely later than for the bottom and middle part of the slab. The presented studies confirmed the impact of boundary conditions on early age compressive strength of cast-in-place concrete. Based on conducted investigations, the actual strength of in-place concrete and the possible dates of prestressing were determined.

## 9. Conclusions

This paper provided the guidelines to predict the temperature field in a concrete structure based on limited input data. The proposed solution, taking into account the thermal and chemical aspect of the hardening concrete, allowed us to simulate successfully the temperature changes in cast-in-place concrete of box bridge girder. The main results of the experimental and numerical studies could be summarized as follows:

- For on-line monitoring of concrete structures, the numerical model of the hardening concrete must be relatively simple, so that it can be an integral part of the SHM system.
- To register temperature of cast-in-place concrete it was suggested to use 1-wire digital sensors and install them on one measuring strip.
- The performed tests of cubic specimens curing under isothermal and semi-adiabatic conditions demonstrated the potential of experimental identification of the adiabatic hydration curve and thus model parameters: $\kappa /n_{0}$, $\bar{n}$ and $A_{0}/\kappa$. In order to increase the quality of determined parameters, it was recommended to perform both experiments, for two mentioned curing conditions.
- The generated temperature of young concrete depends on the element dimensions and the weather conditions. Therefore, two novel nomograms for determination $\kappa /n_{0}$ as a function of an ambient temperature and $\bar{n}$ as a function of an element thickness were developed. An own proposition was dedicated for elements made of high-performance concrete with the thickness range of 10–100 cm.
- The boundary conditions were responsible for the heat exchange with the environment and had a huge impact on the rate gain of concrete temperature, the temperature drop and on the extreme temperature value. Therefore, the suggestions of connective heat transfer coefficient for the concrete surface with a formwork layer $\alpha_{s}^{form}$ and for forced convection $\alpha_{fc}^{}$ were proposed.
- To reliably forecast thermal changes in concrete in a multi-segment structure, it was necessary to carry out preliminary tests on the starting section. The temperature should be measured inside the concrete element and on the formwork surface. Additionally, the ambient temperature as well as the wind speed needs to be registered.
- The numerical evolutions of temperature of high-performance concrete were positively verified in experimental measurements. The daily fluctuations of ambient temperature improved the accuracy of estimated temperature, however the assumption of constant air temperature provided very good results.
- The considered bridge deck proved that the FDM method was appropriate for structural elements with clearly defined boundary conditions as well as quite regular and simple geometry (e.g., concrete slabs and walls). In the case of more complex geometry, the calculations of the temperature evolution should be performed using the finite element method.
- The maturity method gave possibilities to estimate compressive strength distribution with an indication of time to achieve a required strength. The reduced air temperature resulted in a lower initial temperature of the mixture and a lower self-heating temperature, which delayed the development of the concrete strength, which was clearly visible for section No. 3.18.
- The knowledge of the temperature and strength development of concrete before the starting construction process allowed us to select temperature measurement points, to modify the designed concrete mixture and to take proper care of early-age concrete.

## Figures and Tables

**Table 1:** Chemical and physical properties of the cement.

Component	CEM I 52.5 N
SO_3_	2.6%
Cl^-^	0.07%
Na_2_O_eq_	0.5%
Al_2_O_3_	3.9%
C_3_A	1.8%
C_4_AF + 2C_3_A	18.9%
Bulk Density	3.2 g/cm^3^
Blain area	3789 g/cm^2^
Initial setting time	204 min.
Final setting time	257 min.

**Table 2:** Thermophysical properties of concrete C 60/75 class.

C	ρ	$E_{a}/R$	w/c	$\xi_{\max}$	$Q_{\max}$	c	λ
(kg/m^3^)	(kg/m^3^)	(K)	(–)	(–)	(kJ/kg)	(kJ/(kg·K))	(W/(m·K))
440	2570	4620	0.325	0.65	330	0.84	2.0

**Table 3:** Characteristic temperature values.

	$T_{0}$	$T_{env}$	$T_{\max}$	$t_{\max}$	ΔT	$\Delta T/t_{\max}$
Conditions	(°C)	(°C)	(°C)	(h)	(°C)	(°C /h)
**Isothermal**	24.0	24.0	29.2	16.5	5.2	0.32
**Semi-adiabatic**	23.2	24.0	39.6	20.0	16.4	0.82

**Table 4:** Model parameters according to Martinelli et al. [9].

a	b	$\alpha_{s}$	$\alpha_{nc}^{w}$
(–)	(–)	(W/(m^2^·K))	(W/(m^2^·K))
15.5	2.3	3.0	20.0

**Table 5:** Thermophysical parameters—bottom slab, 93 cm thick, stage I.

$\kappa /n_{0}$	$\bar{n}$	$A_{0}/\kappa$	$T_{0}$	$T_{env}^{10\;days}$	$t_{styr}^{}$	$\alpha_{s}^{styr}$	$\alpha_{fc}^{}$	$\alpha_{s}^{form}$
(1/h)	(–)	(–)	(°C)	(°C)	(h)	(W/(m^2^·K))	(W/(m^2^·K))	(W/(m^2^·K))
7.1·10^6^	2.7	1·10^−5^	26.7	22.1	23 – 94	0.40	12.6	2.2

**Table 6:** Thermophysical parameters—web, 40 cm thick, stage II.

$\kappa /n_{0}$	$\bar{n}$	$A_{0}/\kappa$	$T_{0}$	$T_{env}^{10\;days}$	$\alpha_{s}^{form}$
(1/h)	(–)	(–)	(°C)	(°C)	(W/(m^2^·K))
7.6·10^6^	4.3	1·10^−4^	29.1	20.1	2.6

**Table 7:** Thermophysical parameters—top slab, 56 cm thick, stage II.

$\kappa /n_{0}$	$\bar{n}$	$A_{0}/\kappa$	$T_{0}$	$T_{env}^{10\;days}{}_{}$	$\alpha_{fc}^{}$	$\alpha_{s}^{form}$
(1/h)	(–)	(–)	(°C)	(°C)	(W/(m^2^·K))	(W/(m^2^·K))
7.6·10^6^	3.8	1·10^−4^	28.5	20.1	6.5	2.6

**Table 8:** Thermophysical parameters—web, 40 cm thick, stage III.

$\kappa /n_{0}$	$\bar{n}$	$A_{0}/\kappa$	$T_{0}$	$T_{env}^{10\;days}$	$\alpha_{s}^{form}$
(1/h)	(–)	(–)	(°C)	(°C)	(W/(m^2^·K))
12.0·10^6^	4.3	1·10^–4^	14.8	4.4	5.6

**Table 9:** Thermophysical parameters—bottom slab, 80 cm thick, stage I.

$\kappa /n_{0}$	$\bar{n}$	$A_{0}/\kappa$	$T_{0}$	$T_{env}^{10\;days}$	$t_{styr}$	$\alpha_{s}^{styr}$	$\alpha_{fc}$	$\alpha_{s}^{form}$
(1/h)	(–)	(–)	(°C)	(°C)	(h)	(W/(m2·K))	(W/(m2·K))	(W/(m2·K))
7.1·106	3.1	1·10^−5^	26.7	22.1	23 - 94	5.4	18.6	2.2

**Table 10:** Thermophysical parameters—bottom slab, 35 cm thick, stage III.

$\kappa /n_{0}$	$\bar{n}$	$A_{0}/\kappa$	$T_{0}$	$T_{env\_t}^{10\;days}$	$T_{env\_b}^{10\;days}$	$\alpha_{s}^{form}$	$\alpha_{s}^{conc}$
(1/h)	(–)	(–)	(°C)	(°C)	(°C)	(W/(m^2^·K))	(W/(m^2^·K))
12.0·10^6^	4.5	1·10^−4^	14.8	11.3	4.4	5.6	4.0

**Table 11:** Thermophysical parameters—top slab, 57.2 cm thick, stage III.

$\kappa /n_{0}$	$\bar{n}$	$A_{0}/\kappa$	$T_{0}$	$T_{env\_t}^{10\;days}$	$T_{env\_b}^{10\;days}$	$\alpha_{fc}^{}$	$\alpha_{s}^{conc}$
(1/h)	(–)	(–)	(°C)	(°C)	(°C)	(W/(m^2^·K))	(W/(m^2^·K))
12.0·10^6^	3.8	1·10^−5^	14.8	4.4	11.9	10.3	4.0
